# Supplementary material for: Intermittent Bictegravir/Emtricitabine/Tenofovir Alafenamide Treatment Maintains High Level of Viral Suppression in Virally Suppressed People Living with HIV
Source: J Pers Med. 2023 Mar 27;13(4):583. doi: 10.3390/jpm13040583 (PMC10145141; doi:10.3390/jpm13040583)

Supplementary Figure S1. Plasma C_{24h} (24h after the last drug intake) and C_t (3 days or 2 days post-dose, at the end of the discontinuation window) of bictegravir (BIC), emtricitabine (FTC) and tenofovir (TFV) determined in 38 patients.

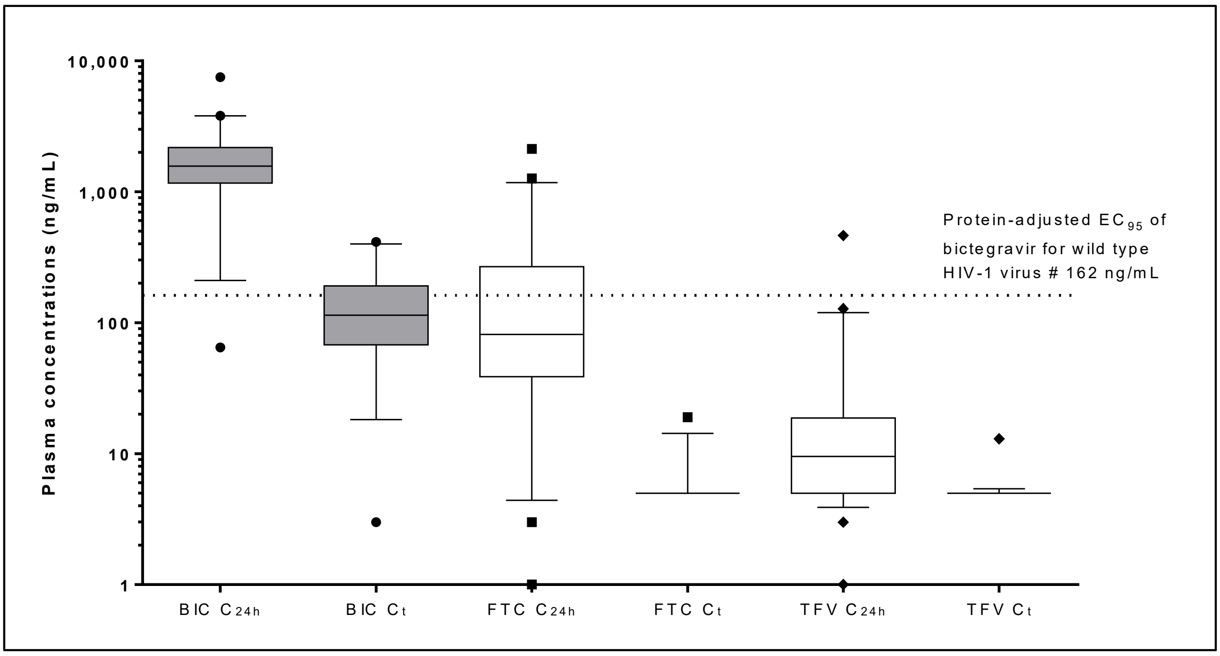

Supplement: Supplementary file 1 [file jpm-13-00583-s001.zip › jpm-2242162-supplementary.pdf]
